# A comprehensive review redefining viruses as therapeutic agents in cancer treatment

**DOI:** 10.1007/s12672-025-03973-3

**Published:** 2025-11-28

**Authors:** Anjana Jadhav, Aravind J, Trupti Patel

**Affiliations:** 1 Professor & Head of Department, Department of Integrative Biology, School of Bio-Sciences & Technology, Vellore Institute of Technology, Vellore, 632014 Tamil Nadu India; 2 Department of BioSciences, School of Bio-Sciences & Technology, Vellore Institute of Technology, Vellore, 632014 Tamil Nadu India

**Keywords:** Oncolytic viruses, Oncolytic virotherapy, Zika virus, PVNPs, Cancer stem cells, Tumors, Strategies, Clinical trials

## Abstract

Oncolytic viruses (OVs) possess the distinctive capability to selectively eliminate malignant cells while exerting reduced impact on healthy tissues compared to traditional therapies. This unique attribute renders them valuable candidates for the development of anti-cancer therapeutics. The recognition of OVs dates to historical observations of tumor regression associated with systemic viral infections. Current advancements in technology enable us to harness this knowledge for therapeutic purposes. However, owing to their inherent pathogenic nature, OVs require genetic modifications to attenuate their virulence, heighten their specificity, and enhance their immunogenic properties. Within the scope of this review, we explore a range of strategies aimed at augmenting the therapeutic potential of OVs. Furthermore, we underscore the synergistic advantages achieved by combining OVs with other cancer treatment modalities. A detailed elucidation of the oncolytic mechanism executed by OVs is provided, and brief mentions are made regarding the positive outcomes observed in clinical trials involving selected OVs. Recent progress in oncolytic viral immunotherapy, exemplified by the utilization of non-infectious Plant virus-based Nanoparticles (PVNPs) in mammals and the application of the Zika virus in glioblastoma treatment, heralds a promising era for innovative therapeutic approaches in the battle against cancer.

**Graphical Abstract:**

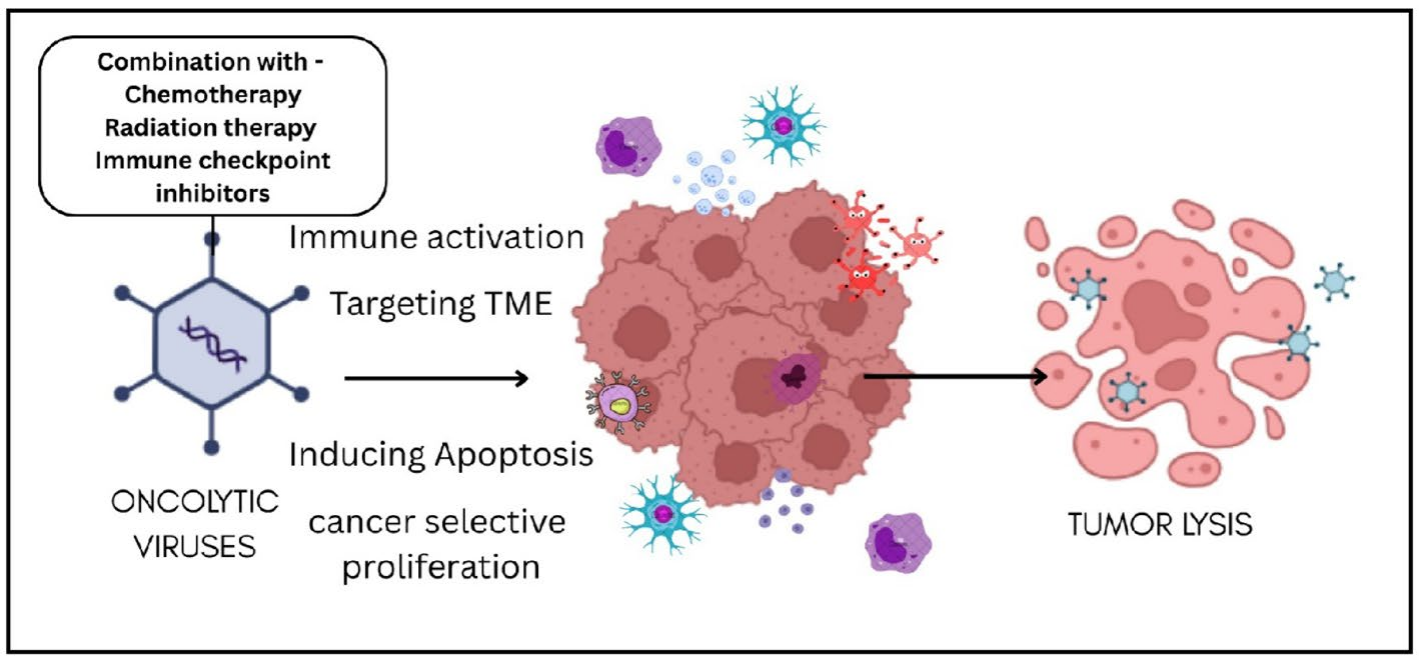

## Article Highlights

- OVs are experiencing renewed interest for their ability to selectively target and destroy cancer cells while sparing healthy tissues.
- Genetic engineering advancements enable precise modifications, enhancing OV safety, specificity, and immune-stimulating properties.
- OVs demonstrate synergistic effects when combined with traditional therapies, offering promising outcomes in various cancer types.
- Ongoing clinical trials are evaluating the safety and effectiveness of oncolytic virotherapies, with some showing encouraging results in terms of tumor reduction and patient survival rates.
- Novel applications, including plant virus-based nanoparticles and the Zika virus in brain cancer therapy, highlight innovative directions in OV research.
- Synthesizing next generation of OVs using directed evolution method.

## Historical perspective

**Fig. 1:**
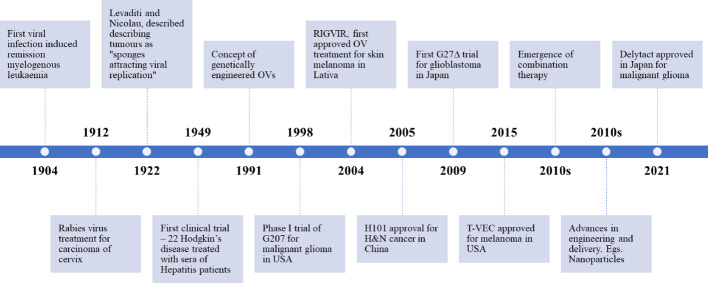
Milestones of oncolytic virus therapy development [1–8]. The figure was created using BioRender (https://www.biorender.com/)

The concept of OVs is not novel but has existed for over a century. (Fig.1) illustrates the key milestones in oncolytic virotherapy. Historical records from the nineteenth century document rare instances where cancer patients experienced clinical remissions following natural infections, a phenomenon once known as the “Saint Peregrine tumor.” Saint Peregrine is considered the patron saint of cancer patients after experiencing a miraculous remission from a leg tumor following an infection, just before a scheduled amputation. This occurred even before the scientific discovery of viruses. The first documented case of viral-induced cancer remission was reported in 1904, in which a 42-year-old woman with myelogenous leukaemia experienced temporary improvement after a presumed influenza infection [1]. In 1922, Levaditi and Nicolau, while developing a smallpox vaccine using vaccinia virus, observed that these viruses demonstrated selective affinity for cancerous tissues. They noted that viruses replicated more vigorously in malignant tissues than in healthy ones, describing tumors as “sponges attracting viral replication“ [2]. The first clinical trial was conducted in 1949, when 22 patients suffering from Hodgkin’s disease were treated with sera and tissue samples from hepatitis patients, thus establishing the oncolytic potential of viruses [3]. However, these early treatments only induced temporary remissions rather than complete recoveries. The field subsequently experienced a setback during the 1960–1970 s due to concerns about uncontrolled infections and insufficient specificity in viral cancer treatments [9, 10]. The renaissance began in 1991 with the advent of recombinant DNA technology and genetic engineering, enabling the development of safer and more specific OVs [11]. A significant milestone occurred in 2004 when RIGVIR, a genetically unmodified ECHO-7 strain enterovirus, became the first oncolytic virus approved by Latvia’s State Agency of Medicines for treating skin melanoma [5]. The world’s first commercialized OV product, Oncorine (H101), a genetically modified adenovirus developed by Shanghai Sunway Biotech Co., Ltd. since 1999, received approval from the Chinese SFDA in November 2005 for treating nasopharyngeal carcinoma in combination with chemotherapy [6]. In 2015, talimogene laherparepvec (T-VEC), an oncolytic herpes virus, made an impact as the first oncolytic virus approved by both the FDA and European Union for treating advanced inoperable melanoma [7]. Since these breakthroughs, substantial research continues to flourish in this field, focusing on developing novel OVs, enhancing their specificity, and exploring innovative combinatorial therapeutic approaches that may revolutionize cancer treatment in the coming decades.

## Introduction

The concept of OVs is not novel but has existed for over a century. Cancer is characterized by the uncontrolled proliferation of abnormal cells. In 2020, it accounted for approximately 10 million deaths worldwide, establishing it as one of the most formidable life-threatening conditions globally. While a multitude of cancer treatment approaches are available, there is a pressing need for more precise strategies that minimize harm to healthy cells. Oncolytic virotherapy has emerged as a promising option for cancer treatment due to its inherent ability to specifically target tumor cells. Genetically engineered OVs were developed to reduce the risk of off-target or uncontrolled viral replication, thereby enhancing their safety profile. These viruses were initially modified to augment tumor specificity and tropism, and later, transgenes were introduced to activate immune responses against tumors or aid in tumor identification [12]. Oncolytic virotherapy holds significant promise due to its capacity for precise destruction of cancer cells, selective replication within tumors, stimulation of the immune system, and systemic therapeutic effects [13]. Table 1 lists various viruses of animal origin used in oncolytic virotherapies. Oncolytic virotherapy can be synergistically combined with other treatment modalities such as chemotherapy, surgery, radiation, hormonal therapy, and immune checkpoint inhibitors to enhance its effectiveness. Clinically successful oncolytic viruses, including H101, T-VEC, G47Δ, OH2, T3011, and Pelareorep, have demonstrated substantial anti-tumor activity across various cancer types, with minimal adverse events [14].

**Fig. 2:**
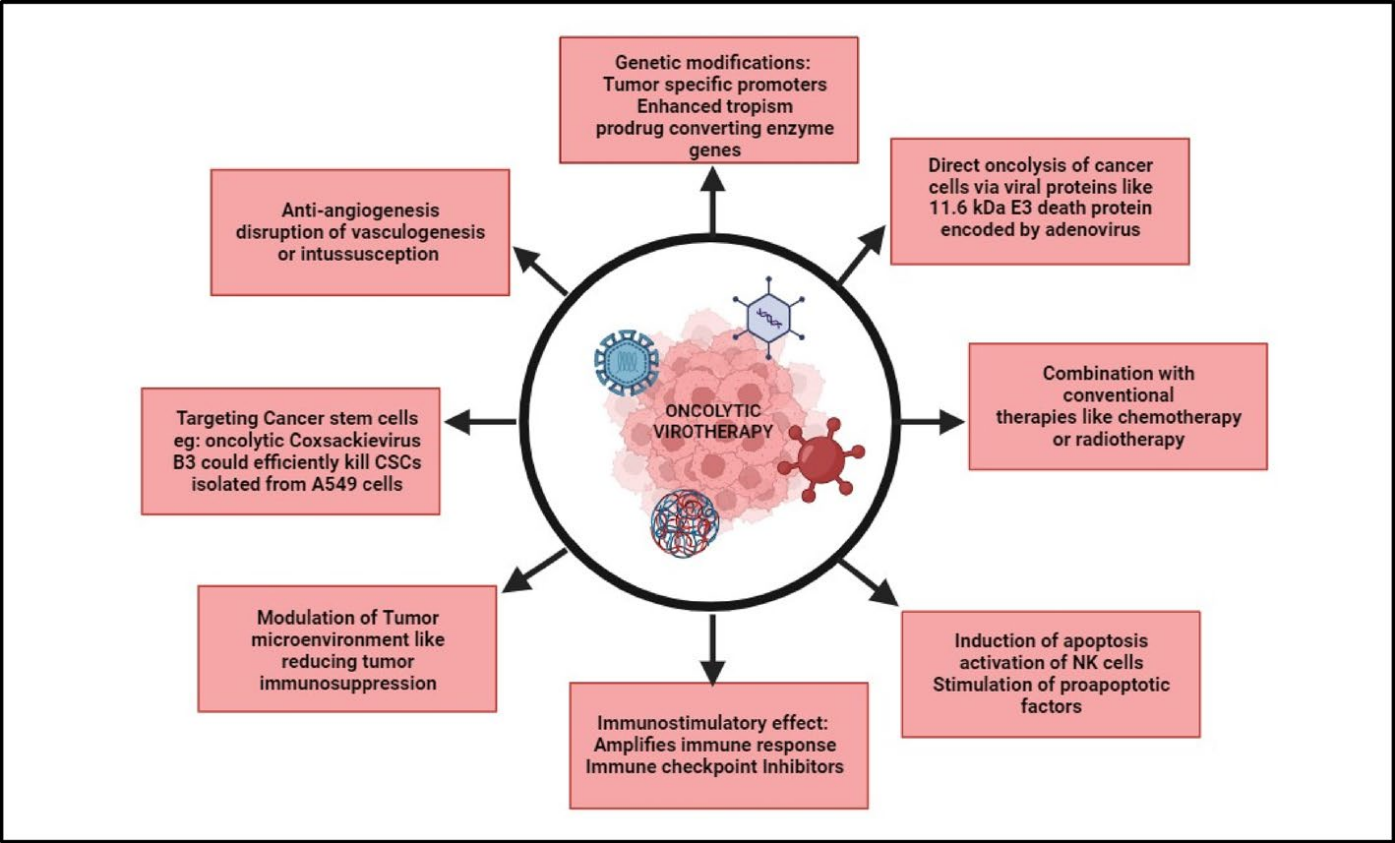
Mechanisms and Therapeutic Strategies of Oncolytic Virotherapy in Cancer Treatment: From Direct Lysis to Immune Modulation. The figure was created using BioRender (https://www.biorender.com/)

This review offers an in-depth examination of various OVs, detailing their types and mechanisms (Fig. 2). It explores the different modification strategies used to boost the oncolytic potential of these viruses and discusses the challenges that must be addressed.

**Table 1:** Various animal viruses used in oncolytic virotherapy

Oncolytic Virus	Type of Virus	Mechanism of action	Against cancer type	Phase of development	Clinical Trial ID	References
T-VEC	Genetically engineered herpes simplex virus type 1 (HSV-1)	Stimulates granulocyte-macrophage colony-stimulating factor (GM-CSF) and enhances local and systemic antitumor immune responses	Melanoma	Listed drugs (FDA approved)	NCT01740297	[15]
H101	Recombinant human adenovirus type 5	Activated host immune system and enhanced cell-medicated immune responses	Oesophageal carcinoma, nasopharyngeal carcinoma, lung carcinoma, and liver carcinoma	Listed drugs (FDA approved)	NCT06031636	[16]
G47Δ	Recombinant oncolytic herpes simplex virus type 1	G47Δ facilitates the priming of the immune system with cancer neoantigens, serving as a platform to revert immunologically “cold” tumors into “hot” tumors.	Malignant glioma, prostate cancer, malignant pleural mesothelioma, recurrent olfactory neuroblastoma	Listed drugs (FDA approved)	UMIN000002661	[17]
CVA21	Coxsackievirus A21	Attaches to ICAM-1 and DAF on cancer cells, enters, replicates, lyses, and spreads, activating immune response.	Melanoma	Phase I-II clinical trial	NCT02316171	[14]
JX-594,	Genetically modified vaccinia virus	Selectively destroys cancer cells through replication-dependent cell lysis and stimulation of antitumoral immunity.	Colorectal, refractory renal cell carcinoma	Phase II-III clinical trial	NCT01171651	[18]
Pelareorep	Reovirus Type 3 Dearing strain	Targets RAS-activated cancer cells, evades immune response, triggers anti-cancer immunity.	Breast cancer, colorectal. Head and neck cancer, Pancreatic cancer	Phase I-II clinical trial	NCT04445844	[19]
KH901	Conditionally replicating oncolytic adenovirus	Lyses telomerase-positive tumor cells and expresses granulocyte macrophage colony-stimulating factor (GM-CSF)	Head and neck cancer	Phase I-II clinical trial	NCT06264453	[20]
T3011	Recombinant HSV-1 oncolytic virus	T3011 virus with IL-12 and anti-PD-1 enhances immune response, inhibits tumor growth	Melanoma	Phase I-II clinical trial	NCT05602792	[21]
Toca 511	Retroviral replicating vector (RRV) based on an amphotropic mouse gamma-retrovirus	Triggers immune response	Glioma, breast cancer	Phase III clinical trial	NCT02598011	[22]
OH2	Genetically engineered oncolytic herpes simplex virus type 2	Selectively amplify in tumor cells and express granulocyte-macrophage colony-stimulating factor to enhance antitumor immune responses	Glioblastoma	Phase III clinical trial	NCT05235074	[23]

## Overview of oncolytic viruses and mechanisms of certain viruses to target oncogenic cells

Viral oncolysis refers to the destruction of tumor cells via selective viral infection, replication, cell lysis, and the subsequent spread of new viral particles within the tumor. Unlike viral gene therapy, where genes are the therapeutic agents, in this approach, the virus itself serves as the treatment. The anti-cancer properties of viruses have been studied for nearly a century, beginning with early research on HSV-1 [11]. OVs are a promising class of anti-cancer agents with a unique ability to selectively infect and destroy cancer cells while largely sparing normal tissues. These viruses can be broadly classified into natural OVs, which have an inherent ability to target and kill cancer cells, and engineered OVs, which are genetically modified to enhance safety, selectivity, and efficacy [24]. This selectivity arises from similarities between the cellular environments of viral infection and malignancy. Cancer cells often exhibit altered signaling pathways, weakened antiviral responses, and disrupted apoptotic mechanisms, making them more vulnerable to viral infection and replication. Some naturally occurring tumor-killing viruses include the reovirus, Newcastle disease virus, and Coxsackievirus A21.

## Mechanisms of selectivity in oncolytic viruses

**Fig. 3:**
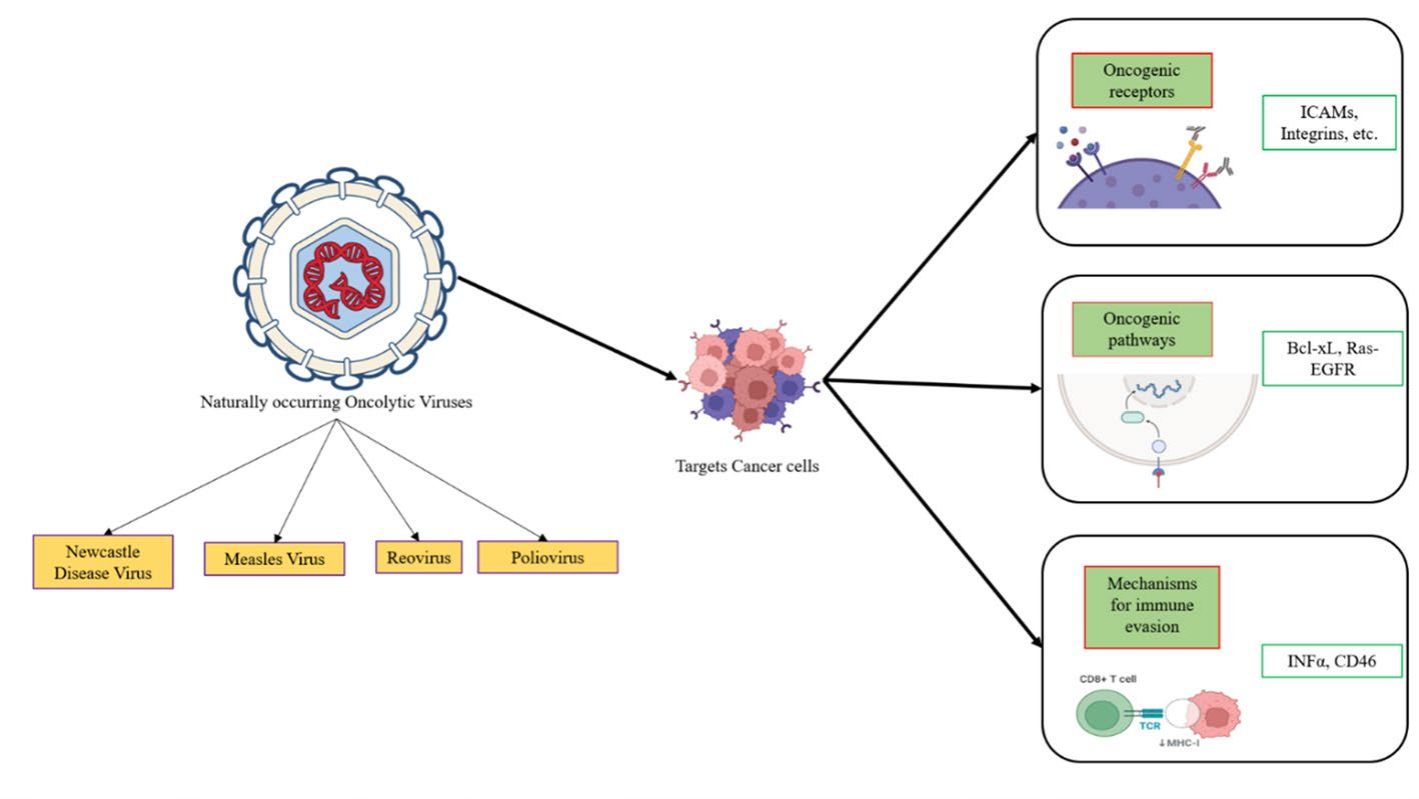
How Natural Oncolytic Viruses Strategically Target Cancer Cells? Naturally occurring oncolytic viruses selectively target cancer cells by exploiting specific mechanisms:1. Oncogenic Receptors: Overexpressed receptors like ICAMs and integrins facilitate viral entry into cancer cells. 2. Oncogenic Pathways: Deregulated pathways such as Bcl-xL and Ras-EGFR are utilized to support viral replication. 3. Immune Evasion Mechanisms: Viruses bypass host immunity by interfering with molecules like IFN-α and targeting CD46, often upregulated in cancer cells. The figure was created using BioRender (https://www.biorender.com/)

Cancer cells actively rewire transcriptional and signaling networks to promote survival, immune evasion, and metastasis. Viruses exploit similar pathways for replication, creating a natural susceptibility in cancer cells due to overlaps between carcinogenic processes and viral infection. Tumor cells serve as ideal targets for OVs due to their rapid proliferation and elevated metabolic activity compared to normal cells. OVs leverage three primary mechanisms to target cancer cells: interaction with oncogenic receptors, immune evasion, and selective targeting of oncogenic pathways (Fig. 3).

### Interaction with oncogenic receptors

Viral infection of cancer cells begins with the virus’s ability to recognize and enter suitable cellular targets, primarily via cell surface receptors. Many OVs are selected for their ability to recognize and bind specific cell surface receptors or molecules that are frequently overexpressed on cancer cells compared to normal cells. These receptors act as entry points for the virus, which binds selectively to receptors such as CAR, laminin, CD155, and CD46, commonly overexpressed on cancer cells. This increased expression allows Adeno - [25], Sindbis [26], Polio [27], and the Measles virus [28] to enter and infect cancer cells more efficiently. For example, the measles virus (Edmonston strain) targets CD46, a receptor overexpressed by malignant cells to evade the complement system, facilitating selective tumor cell destruction. Similarly, the elevated levels of ICAM-1 (Intercellular Adhesion Molecule – 1) and decay-accelerating factor (DAF/CD55) in cancers like breast cancer, multiple myeloma, and melanoma allow coxsackievirus (e.g., coxsackievirus A21) to induce oncolysis within these malignancies. The Vaccinia virus, part of the poxvirus family, has emerged as a promising OV for targeting cancer cells. This potential arises from its ability to selectively infect cells that overexpress the Epidermal Growth Factor Receptor (EGFR), a receptor commonly found in high amounts on tumor cells. By attaching to EGFR, the Vaccinia virus enhances its entry, replication, and spread within malignant cells through the EGFR-RAS signaling pathway. This receptor-targeted entry mechanism supports viral proliferation and facilitates immune evasion and oncolytic effects, contributing to its effectiveness in cancer treatment.

### Selective targeting of oncogenic pathways: a brief insight into the mechanism of Newcastle disease virus

Cancer cells have been shown to circumvent apoptosis by increasing the expression of anti-apoptotic molecules, particularly those belonging to the Bcl family. This heightened expression of anti-apoptotic factors renders cancer cells optimal targets for specific OVs. NDV is a unique OV that selectively targets cancer cells exhibiting high levels of Bcl-xL expression. Bcl-xL, an anti-apoptotic protein from the Bcl-2 family, is commonly upregulated in cancer cells. This allows them to evade programmed cell death and survive under conditions that would typically trigger apoptosis in healthy cells. This overexpression of Bcl-xL makes cancer cells more susceptible to NDV infection and replication. NDV exploits this vulnerability by selectively infecting these cancer cells, which not only provide a permissive environment for viral replication but also enable the virus to proliferate within the tumor without being halted by the usual apoptotic defenses. Once inside the cancer cell, NDV initiates its replication cycle, producing viral particles that accumulate within the cell. NDV triggers the formation of syncytia, large multinucleated cells formed by the fusion of NDV-infected cells with their neighbors. Syncytia formation is particularly advantageous for NDV, as it facilitates viral spread across multiple cancer cells without requiring extracellular release. This enables the virus to evade immune detection and continue replicating within the tumor microenvironment (TME). This cell fusion and syncytia formation are critical for NDV’s survival and propagation, as the infected syncytial cells serve as hubs of viral production, continually spreading the virus throughout the tumor. The destruction of cancer cells through syncytial lysis, along with the release of tumor antigens, alerts the immune system and triggers an inflammatory response that enhances tumor recognition and targeting. Through this mechanism, NDV not only induces direct oncolysis in Bcl-xL-overexpressing cancer cells but also leverages the high Bcl-xL expression of these cells to sustain its own replication [29].

### Circumventing immune responses: reovirus exploits Ras signaling for oncolysis

The Ras signaling pathway is frequently altered in cancer cells, impacting the regulation of cell death and proliferation. Reovirus and vaccinia virus are classified as natural OVs due to their selective targeting of cancer cells with active Ras signaling. Reovirus preferentially replicates in Ras-transformed cells. The pathway begins with the virus attaching to host cells via junctional adhesion molecule-A (JAM-A) and sialic acid residues, enabling internalization through receptor-mediated endocytosis. Once inside, acidification and proteolytic processing by endosomal cathepsins transform the intact virion into an infectious subviral particle (ISVP), which penetrates the endosomal membrane to release the viral core into the cytoplasm. This core acts as a protected factory for transcription, producing capped positive-sense mRNAs that exit into the cytosol for translation of viral proteins. In healthy cells, the detection of viral double-stranded RNA (dsRNA) PKR leads to phosphorylation of eIF2α, shutting down global protein synthesis, while RIG-I and MDA5 pathways drive robust type I interferon responses. These mechanisms collectively restrict viral replication [30]. However, in Ras-activated cancer cells, where Ras effector pathways such as Raf/MEK/ERK are chronically active, PKR activation is impaired, preventing eIF2α phosphorylation and allowing unhindered translation of viral mRNAs. Simultaneously, reduced IFN (interferon) signaling further enhances permissiveness. This selective vulnerability is the cornerstone of reovirus oncolytic therapy. Inside tumor cells, newly synthesized structural proteins and genomes assemble in inclusion bodies where progeny virions are packaged. These mature reovirus particles accumulate until they ultimately trigger cell lysis, releasing thousands of infectious units that propagate to neighboring cancer cells, amplifying oncolysis within the tumor [31]. Moreover, the lysis of infected cells releases tumor neoantigens that can prompt an immune response, further targeting and attacking the tumor [32]. Beyond direct cytolysis, reovirus infection provokes local immunogenic cell death, enhancing tumor antigen release and promoting dendritic cell activation. This fosters a systemic anti-tumor immune response, broadening the therapeutic impact beyond the initial sites of viral replication.

### Mechanism of oncolysis by coxsackievirus A21: targeting ICAM-1 and DAF-expressing tumors

Two important cell surface molecules, ICAM-1 and DAF, are commonly overexpressed in a variety of cancers, such as glioblastoma, colorectal cancer, triple-negative breast cancer, and melanoma [33, 34]. Through mechanisms of immune evasion, metastasis facilitation, and resistance to complement-mediated cell lysis, these molecules play crucial roles in promoting the progression of tumors. As a ligand for the integrin LFA-1 (lymphocyte function-associated antigen-1), ICAM-1, a member of the immunoglobulin superfamily, allows tumor cells, especially melanocytes, to interact with circulating lymphocytes. In addition to enabling immune evasion, this interaction may facilitate melanoma cell migration across endothelium and metastasis. However, DAF is a complement regulatory protein that prevents C3/C5 convertases from forming, protecting tumor cells from complement-mediated cell destruction. CVA21, a naturally occurring enterovirus most commonly associated with the common cold, finds an ideal receptor complex on the surface of cancer cells when both ICAM-1 and DAF are overexpressed. Target cells are infected by this virus using a two-step receptor-mediated mechanism. By acting as a membrane attachment or sequestration receptor, DAF efficiently concentrates CVA21 on the surface of tumor cells. The virus is brought close to the cell by this binding, but internalization is not made possible by it alone. Rather, ICAM-1 serves as the main entry receptor. The conformational changes in the CVA21 capsid that enable viral uncoating and endocytosis into the host cell depend on its expression [35]. Rapid viral replication, effective internalization, and highly specific viral attachment are all made possible by the coordinated action of DAF and ICAM-1, which leads to tumor cell lysis. Since most healthy tissues do not co-express high levels of both receptors, while many malignant cells do, this dual-receptor mechanism is especially useful for targeting tumors. The clinical significance of this mechanism has instigated an increasing interest in CVA21 as an oncolytic virotherapy molecule. In preclinical models, the potent cytolytic activity of CVA21 against numerous tumor cells that express ICAM-1 and DAF, and the capability to induce immunogenic cell death that can initiate systemic anti-tumor immune responses, have been shown. These encouraging results have progressed CVA21 into several Phase I and II clinical trials [36].

## Genetically modified oncolytic viruses

Genetically modified OVs are essential to improve the safety, specificity, and efficacy of virotherapy. Natural viruses may infect both healthy and cancerous cells, leading to non-specific targeting and potential off-target effects, which can be particularly detrimental in immunocompromised patients. Genetic modifications enhance tumor specificity, allowing OVs to selectively infect and replicate within cancer cells while minimizing damage to normal tissues. Additionally, engineered OVs can express therapeutic genes, boost anti-tumor immunity, and reduce the risk of viral shedding or recombination with wild-type viruses, making them more effective and safer for clinical applications.

Optimizing the spread and delivery of OVs is pivotal for therapeutic efficacy. Numerous host barriers present significant challenges in achieving optimal outcomes with OVs in patients. OVs administered via intra-tumoral (I.T.), intravenous (I.V.), or intramuscular (I.M.) routes often face reduced effectiveness, especially in I.V. delivery, due to neutralizing antibodies and complement proteins in the bloodstream. Consequently, it becomes imperative to formulate strategies that circumvent the neutralization of OVs by antibodies and complement proteins within the circulatory system. Classical strategies for shielding oncolytic vectors from neutralization include the exchange of envelope proteins within a virus species or among related virus families, the incorporation of multiple epitope replacements, the development of cell carriers, and chemical modifications [37]. Several classical strategies have been devised for immune detection while preserving tumor-specific targeting. One prominent approach is envelope exchange or pseudo-typing, which involves replacing the viral envelope glycoproteins with those derived from unrelated, non-cross-reactive viruses to evade recognition by existing antibodies. For example, measles virus (MV) glycoproteins have been substituted with glycoproteins from Tupaia paramyxovirus (TPMV) or canine distemper virus (CDV), yielding chimeric viruses that resist neutralization by anti-MV antibodies yet retain their ability to infect target cells. Although this strategy effectively avoids pre-existing immunity, it can sometimes disrupt critical viral functions like membrane fusion or proper particle assembly due to incompatibility between viral components [38].

PEGylation refers to the covalent attachment of polyethylene glycol (PEG) chains to the surface of OVs. This advanced form of surface engineering addresses major challenges associated with systemic OV delivery by improving circulation time and reducing immune-mediated clearance. The addition of PEG creates a hydrophilic and steric shield around the viral capsid or envelope, effectively masking immunogenic epitopes and minimizing recognition by the host immune system. This protective coating prevents complement activation and neutralizing antibody binding, thereby prolonging viral persistence in circulation and increasing the probability of tumor localization [39]. A study by Tesfay et al. demonstrated that in passively immunized mice, PEGylated vesicular stomatitis virus exhibited significantly prolonged circulation times compared to its unmodified form, confirming PEG’s ability to protect viral particles from rapid neutralization by circulating antibodies. Importantly, PEGylation conferred partial immune shielding without entirely compromising viral infectivity. Beyond improving pharmacokinetics, PEGylation also enhances safety by reducing off-target toxicity. Virus-induced hepatotoxicity, evaluated through serum levels of alkaline phosphatase (ALP) and alanine aminotransferase (ALT), revealed that mice treated with unmodified VSV displayed markedly elevated enzyme levels compared to those receiving PEGylated VSV or phosphate-buffered saline (PBS) controls. These findings indicate that PEGylation mitigates hepatic injury and systemic toxicity, likely by restricting viral replication in non-tumor tissues [40].

Another unique method employs cell carriers, making use of immune cells such as monocytes and granulocytes to ferry the OV through the bloodstream. Cell carriers offer an advanced and promising strategy in oncolytic virotherapy aimed at improving the delivery, stability, and therapeutic efficacy of OVs. Among the most widely used carrier cells are T lymphocytes and mesenchymal stem cells (MSCs), both valued for their inherent tumor-homing properties and ability to efficiently load and transport viral particles. Other cell types, such as neural stem cells and monocytes, have also demonstrated potential in enhancing OV bioavailability and tumor specificity in preclinical models [41, 42]. These carriers can either internalize viral particles or bind them to their surface, enabling stable circulation and controlled release within the TME. For instance, studies with reovirus have demonstrated that when carried within blood cell compartments, the virus can traverse the circulatory system intact, preferentially infect tumor tissue, and spare normal organs, despite the presence of antiviral antibodies [43]. Current research is focused on genetically engineering carrier cells to optimize viral payload capacity, targeting precision, and immune compatibility. Furthermore, combinatorial strategies, including the integration of OV-loaded carriers with immune checkpoint inhibitors or adoptive immune therapies (such as CAR-T or CAR-NK cells), are being explored to achieve synergistic and durable antitumor responses, paving the way for next-generation systemic oncolytic virotherapies [44].

In addition, genetic engineering strategies offer nuanced approaches to immune evasion. Incorporating miRNA-responsive elements into viral genomes, such as in modified adenoviruses, allows selective replication in tumor cells by exploiting the absence or low levels of specific microRNAs in cancerous tissues, thereby reducing exposure to systemic immune responses. Similarly, engineering viruses to include specialized 5′-untranslated regions (5′-UTRs), like those responsive to rFGF-2 that require high levels of eIF4E (commonly overexpressed in tumors), restricts viral translation and replication to malignant cells [45]. Together, these approaches help to defend OVs from neutralization, facilitate preferential destruction of cancer cells, and maintain the effectiveness of oncolytic virotherapy even in individuals with strong antiviral immunity.

Different types of viruses exhibit varying degrees of natural affinity and preferential replication tendencies in distinct tumor cell types. Genetically engineered OVs are specifically designed to enhance their selectivity for targeting tumors (Table 2). Two primary modification strategies are employed to improve the precision of OVs in tumor targeting. The first strategy involves augmenting the affinity and binding activity of viruses towards the overexpressed receptors on the surface of tumors. The increasing identification of tumor-specific receptors or antigens provides OVs with additional promising avenues for enhancing targeting accuracy. Another effective approach to refining tumor targeting involves enhancing the replication capacity of OVs. Some viruses possess inherent mechanisms that promote replication, while molecular engineering techniques enable the modification of viruses to enhance their replication efficiency specifically within cancer cells. It has been postulated that the loss of tumor-suppressor genes and dysregulation of signalling pathways within tumor cells can further contribute to the selective replication of OVs within tumors. Another strategy involves integrating so called “suicide genes” into the OVs. These genes sensitize tumor cells to externally administered prodrugs or initiate cell death through alternative mechanisms. Such genetic elements may encompass enzymes that heighten cytotoxicity or transform otherwise inert prodrugs into potent cytotoxic agents exclusively within cells expressing the self-destruction gene. OVs can be engineered to carry genes encoding major cytokines such as IL-2, IL-10, TNF-α, and thymidine kinase, which are often suppressed in cancer.

The immune system, however, plays a dual role in oncolytic virotherapy, acting both as a facilitator of antitumor immunity and as a barrier that limits viral efficacy. On one hand, OVs can enhance antitumor responses by inducing immune mediated cell death in infected tumor cells, thereby releasing tumor-associated antigens (TAAs), pathogen-associated molecular patterns (PAMPs), and danger signals (DAMPs). These molecules recruit and activate APCs, such as dendritic cells, which prime CTLs and promote systemic antitumor immunity. Lytic infection further attracts immune effector cells, including NK cells and macrophages, to the TME, directly killing tumor cells. But on the other hand, the immune system can hinder OV efficacy through rapid antiviral responses. Neutralizing antibodies, complement proteins, and cytotoxic T cells can clear viral particles or infected cells before sufficient tumor infection occurs. Pre-existing immunity to the viral strain, due to prior vaccination or infection, further accelerates viral clearance. In some cases, excessive antiviral activation may trigger harmful inflammation or systemic toxicity, limiting safe dosing. Balancing these opposing effects is central to optimizing the therapeutic potential of OVs and several relevant strategies to achieve this will be discussed in detail ahead.

**Table 2:** Genetic modification strategies in oncolytic virus engineering

Modification Strategy	Purpose	Examples of Viruses	Mechanism	References
Deletion of virulence genes	Increases tumor selectivity by disabling replication in normal cells	HSV-1 (ICP34.5, ICP6), Adenovirus (E1B-55 K)	Targets tumors with defective antiviral pathways (e.g., p53, PKR)	[46, 47]
Insertion of therapeutic transgenes	Enhances immune response or therapeutic effect	HSV-1 (GM-CSF in T-VEC), VSV (IL-12), VACV (anti-PD-L1)	Express cytokines, checkpoint inhibitors, or prodrug-converting enzymes	[46, 48, 49]
Promoter substitution with tumor-specific promoters	Restricts viral gene expression to cancer cells	Adenovirus (hTERT, E2F1), HSV (Nestin)	Drives selective viral replication based on tumor-specific transcriptional activity	[50–52]
Capsid/Envelope modification (retargeting)	Alters viral tropism to improve tumor cell targeting	Adenovirus (RGD motif), Measles virus (EGFR targeting), AAV (peptide ligands)	Enhances binding to receptors overexpressed in tumors	[53–55]
MicroRNA target site insertion	Prevents replication in normal tissues via post-transcriptional regulation	VSV, Adenovirus, HSV	miRNA target sites restrict gene expression in non-cancerous cells	[56–58]
Immune evasion modifications	Prolongs viral persistence by avoiding rapid immune clearance	VACV (E3L, B18R), Measles virus	Encodes immune-modulating proteins or downregulates MHC, IFN responses	[59]
Incorporation of suicide genes	Allows safety control via inducible cell death	HSV (TK), Adenovirus (CD/5-FC system)	Converts non-toxic prodrugs into toxic metabolites in infected cells	[60, 61]
Enhanced fusogenicity genes	Promotes syncytia formation and direct tumor killing	Measles virus, NDV	Increases oncolysis via membrane fusion	[62, 63]
Arming with bispecific T-cell engagers (BiTEs)	Redirects immune cells to tumor antigens	Adenovirus, VACV	Encodes BiTEs that bind tumor antigen and CD3 on T cells	[64]
Arming with anti-angiogenic genes	Disrupts tumor vasculature and inhibits growth	HSV, VACV	Expresses genes like endostatin or angiostatin	[65]
Insertion of reporter genes	Enables imaging, tracking, and dose monitoring	VSV (Luciferase), HSV (GFP)	Allows real-time assessment of viral spread and replication	[66]

### T-VEC (talimogene laherparepvec) – Imlygic^®^

T-VEC, is the first virus approved by the FDA for advanced melanoma in 2015, is administered via intra-lesional injection. T-VEC is a genetically modified Herpes simplex virus-1 (HSV-1) engineered to selectively replicate in cancer cells, enhance anti-tumor immunity, and improve the host’s tumor-fighting potential. T-VEC differs from wild-type HSV-1 through four principal genetic modifications: insertion of the GM-CSF cassette, deletion of infected cell Protein (ICP) 34.5, deletion of ICP47, and early expression of US11 [15]. (i) GM-CSF (Granulocyte-Macrophage Colony-Stimulating Factor) enhances anti-tumor immune responses by recruiting and activating immune cells, (ii) Deletion of ICP34.5 (γ34.5), a virulence factor that inhibits the host PKR antiviral pathway, increases viral selectivity for cancer cells with defective PKR signaling. This deletion also improves immune recognition of infected cells [46], (iii) Deletion of ICP47, a viral protein that inhibits antigen presentation, restores MHC class 1 expression and enhances visibility of infected tumor cells to CTLs, (iv) Early expression of US11, typically expressed late in wild-type HSV-1 infection to counteract PKR, ensures efficient viral replication even in tumors with intact PKR responses broadening the therapeutic scope of T-VEC. This strategically modified virus functions as a potent dual-action therapeutic agent, directly destroying tumor cells while simultaneously stimulating a systemic anti-tumor immune response, representing a significant advancement in cancer immunotherapy for patients with limited treatment options. Clinically, T-VEC has shown encouraging efficacy and safety in advanced melanoma. In the Phase III OPTiM trial, T-VEC achieved a durable response rate (DRR) of 19.3% compared to 1.4% with GM-CSF (*p* < 0.0001) and an overall response rate (ORR) of 31.5%, including 16.9% complete responses (CRs) and 14.6% partial responses (PRs) [67]. Real-world studies further demonstrated higher ORRs of 57% [68], 88.5% [69], and 56.5% [70], with most adverse events being mild, such as fatigue, chills, and flu-like symptoms, and < 2% experiencing grade 3/4 toxicities [67]. These findings reinforce the therapeutic promise of T-VEC as a well-tolerated and effective oncolytic virothrerapy in melanoma.

### Oncorine^®^ (H101)

H101, is an attenuated adenoviral vector approved in China for the treatment of head and neck carcinoma, typically in combination with chemotherapy. The key genetic modifications in H101 are the deletion of E1B-55 K and four strategic deletions in the viral E3 region [71]. The E1B-55 K gene encodes a protein that inhibits the tumor suppressor p53, enabling viral replication in normal cells by evading host antiviral responses. Consequently, H101 can selectively replicate only in p53-deficient cancer cells, sparing normal tissue [47]. The four deletions in the E3 region prevent synthesis of critical proteins: E3-11.6 K, which encodes the adenovirus death protein (ADP) involved in cell lysis and viral release, its deletion restricts viral spread in normal cells. E3-19 K, a protein that suppresses MHC class 1 expression, its deletion enhances immune recognition and promotes CTL mediated clearance of infected tumor cells [72]; E3-10.4 K/14.5 K which form the Receptor Internalization and Degradation (RID) complex; their deletion restores TRAIL- and Fas-mediated apoptosis in infected tumor cells resulting in increased apoptosis of infected tumor cells when deleted; and E3-14.7 K, which blocks TNF-α-induced apoptosis, its deletion sensitizes tumor cells to TNF-mediated cell death. Together, these genetic alterations make H101 a selective oncolytic agent with enhanced immunotherapeutic capabilities, making a major advancement in targeted cancer virotherapy. Clinically, H101 demonstrated a response rate of 30.4%, significantly higher than control lesions (13.0%, *P* < 0.05), and showed improved outcomes when combined with chemotherapy (*P* < 0.001). Patients developing post-injection fever had greater tumor regression (69.2% vs. 21.2%, *P* < 0.005), suggesting immune activation may enhance efficacy. Adverse events, including fever (30.2%), nausea/vomiting (34.0%), and leucopenia (49.1%), were manageable and consistent with OV administration [73]. These findings underscore H101’s potential as a selective and immunogenic oncolytic agent, supporting its further clinical development.

### Delytact (G47Δ)

Teserpaturev (marketed as Delytact) became Japan’s first approved OV-based therapy for glioblastoma treatment in 2021. It is based on a genetically engineered HSV-1 with three key modifications: deletion of ICP47, deletion of ICP34.5, and inactivation of ICP6 via insertion of the *Escherichia coli* lacZ gene. ICP6 encodes the large subunit of ribonucleotide reductase, an enzyme essential for viral DNA replication, and its replacement with the bacterial lacZ gene effectively neutralizes its function. When administered intratumorally to patients with malignant glioma, Delytact exhibits a dual mechanism of action: first, the modified virus selectively replicates within tumor cells and destroys them through the viral replication process, exerting a direct cytocidal effect; second, the treatment induces tumor-responsive T cells, thereby activating antitumor immunity. This combinatorial approach—direct oncolysis coupled with immune stimulation—represents a significant advancement in glioblastoma treatment, potentially extending survival for patients with this aggressive and treatment-resistant malignancy. The therapeutic rationale behind Delytact exemplifies the progress in engineering viruses with enhanced tumor selectivity and immunostimulatory properties, offering new hope in the challenging landscape of brain cancer therapy [74]. In a phase 2 trial, G47Δ achieved a 1-year survival rate of 84.2%, with a median overall survival of 20.2 months and progression-free survival of 4.7 months, notably exceeding outcomes from conventional therapies. Administered intratumorally in up to six doses, it showed long-term survival benefits despite a modest 5.3% radiological response rate, likely due to delayed immunological effects. Adverse events were predominantly immune-related, including fever, headache, and tumor swelling, with only two grade 3 events and no dose-limiting toxicities reported [75]. These findings highlight Delytact as a safe and efficacious therapeutic option for glioblastoma. Table 3 gives a comparative outlook of approved oncolytic virotherapies.

**Table 3:** Comparison of approved oncolytic virotherapies

Feature	T-VEC (Imlygic^®^)	Oncorine^®^ (H101)	Delytact (G47Δ)
Virus Backbone	Herpes Simplex Virus type 1 (HSV-1)	Human Adenovirus type 5	Herpes Simplex Virus type 1 (HSV-1)
Country/Year of Approval	USA (FDA), 2015	China (SFDA), 2005	Japan (PMDA), 2021
Indication	Advanced melanoma	Head and neck squamous cell carcinoma (with chemotherapy)	Glioblastoma
Route of Administration	Intratumoral (intra-lesional) injection	Intratumoral (with chemotherapy)	Intratumoral
Key Genetic Modifications	- Insertion of GM-CSF - Deletion of ICP34.5 and ICP47 - Early expression of US11	- Deletion of E1B-55 K - Deletion of E3 genes (11.6 K, 19 K, 10.4 K/14.5 K, 14.7 K)	- Deletion of ICP34.5 and ICP47 - Inactivation of ICP6 via lacZ insertion
Tumor Selectivity Mechanism	Replicates in cancer cells with defective PKR signaling	Selective replication in p53-deficient tumor cells	Selective replication in tumor cells lacking functional ICP6
Immune Activation Features	GM-CSF expression; restored MHC-I presentation via ICP47 deletion	Enhanced MHC-I expression; restored TNF/Trail-mediated apoptosis	Induces tumor-specific T cells; enhances antigen presentation
Mechanism of Action	Direct oncolysis + systemic immune activation	Selective oncolysis + enhanced CTL-mediated tumor clearance	Direct oncolysis + induction of antitumor T cell responses

## Cancer hallmarks and TME targetable by OVs

**Fig. 4:**
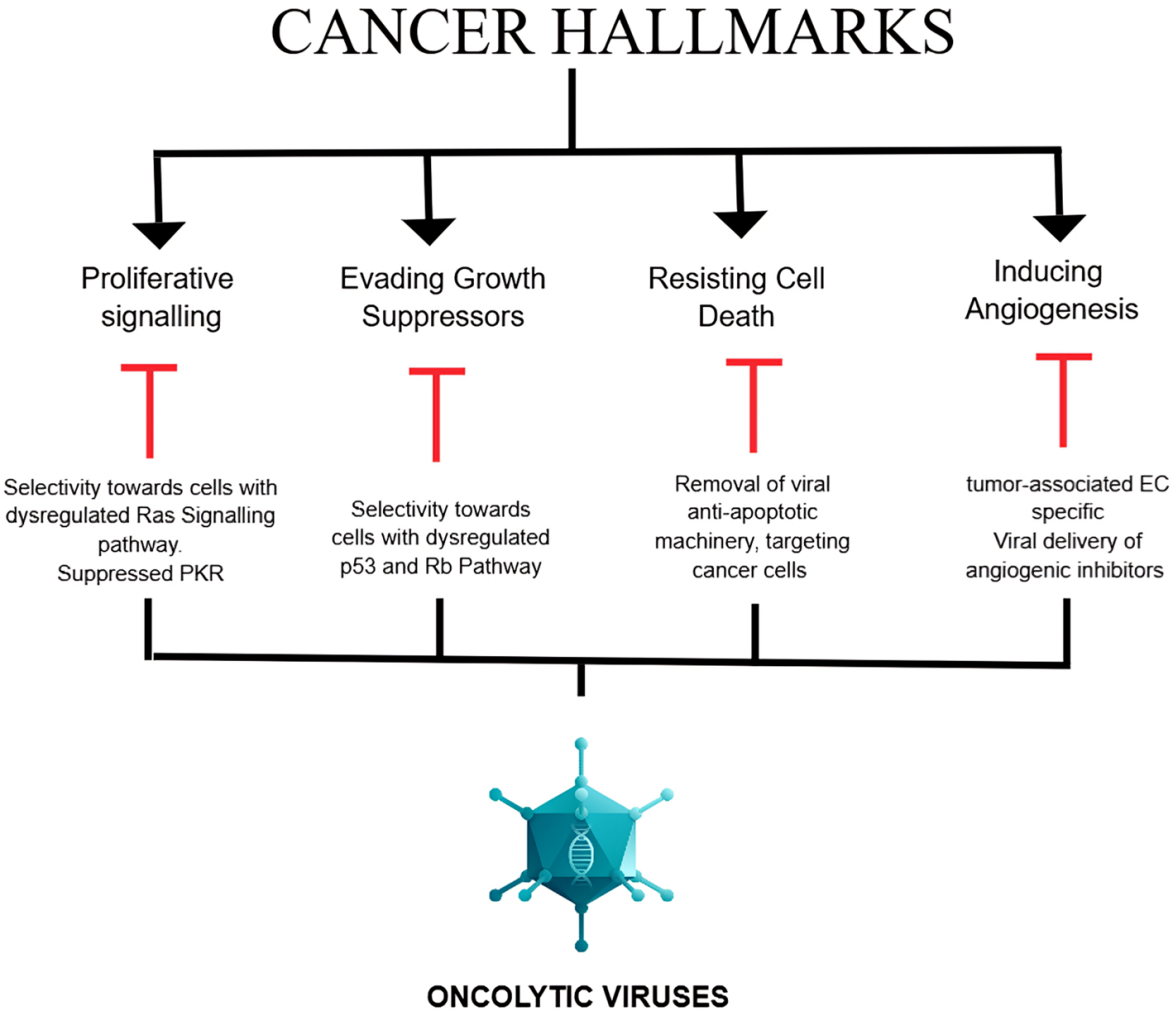
Mechanisms by which oncoviruses suppress cancer hallmarks. The figure was created using BioRender (https://www.biorender.com/)

It is widely understood that cancer has several key characteristics or hallmarks: genomic instability and mutations, uncontrolled growth, evasion of growth suppressors, resistance to cell death, increased inflammation, heightened metabolism, and the remarkable ability to induce angiogenesis, invasion, and metastasis [76, 77]. A tumor is not merely a mass of proliferating cells; it is a complex assembly of infiltrating and resident host cells, secreted substances, and extracellular matrix. Tumor cells induce substantial molecular, cellular, and physical alterations within the host tissues to facilitate their growth and progression. Tumor cells interact bidirectionally with their microenvironment through cell-cell communication and signalling pathways, affecting tumor growth, invasion, and metastasis [78] (Fig. 4).

### Evading growth suppressors

#### p53 suppressor pathway

The p53 tumor suppressor pathway is a network of genes and proteins that controls cell division, DNA repair, and apoptosis to prevent the formation of abnormal cells. In cancer cells, the p53 pathway is often suppressed due to mutations or activation of oncogenes, leading to unchecked cell growth and resistance to cell death [79]. Reoviruses and Parvoviruses typically replicate in cells where the p53 pathway is defective or inhibited, as healthy cells with functional p53 would trigger apoptosis upon viral infection. EIB-55 K, an adenoviral oncoprotein, represses p53 transcriptionally. ONYX-015, a mutant adenovirus lacking EIB-55 K, cannot replicate in normal cells, thus selectively targeting cancer cells [80].

#### Retinoblastoma suppressor pathway

The Retinoblastoma protein (pRB) is crucial for regulating cell proliferation by interacting with the E2F family of transcription factors to control genes essential for cell cycle progression and S phase entry [81]. Transforming proteins from DNA tumor viruses, such as adenovirus E1A and simian virus 40 large T antigen, form complexes with the Rb tumor suppressor protein. The conserved regions 1 and 2 of adenovirus E1A protein bind to Rb, disrupting its interaction with E2F, thereby inactivating the tumor-suppressing function of Rb by interfering with its cellular targets [82]. Mutant adenoviruses with defective E1A can selectively replicate in cells with impaired pRB function. RB1 VCN-01 is an oncolytic adenovirus designed to replicate in tumor cells that exhibit elevated levels of free E2F-1, a result of a dysfunctional pRB pathway [83].

#### Resisting cell death

Apoptosis, a form of programmed cell death, is triggered when cells are damaged or exposed to specific stimuli. Cancer cells frequently evade apoptosis through various mechanisms, including the overexpression of anti-apoptotic proteins like Bcl-xl. The Bcl-2 protein family is among the most well-studied regulators of apoptosis. In the co-evolution of host-pathogen interactions, viruses have also developed strategies to inhibit apoptosis, facilitating their replication. Anti-apoptotic viral proteins, such as v-Bcl2 and v-FLIP from gamma herpesvirus [84], vMIA (viral mitochondria-localized inhibitor of apoptosis), and pUL36 from cytomegalovirus, help viruses evade apoptosis [85]. Deleting these genes in herpesviruses can produce tumor-selective, highly potent OVs. For example, the removal of the anti-apoptotic gene F1L in the Copenhagen strain VACV ΔTK enhances oncolytic activity in glioblastoma models [86].

### Inducing angiogenesis

Angiogenesis refers to the process of forming new blood vessels from existing ones. Endothelial cells (ECs) line blood vessels and facilitate the exchange between the bloodstream and surrounding tissues. Angiogenesis is initiated by the sprouting of endothelial cells in response to angiogenic signals. Some OVs can selectively infect tumor-associated ECs while sparing normal ones [87]. In a murine colorectal carcinoma xenograft model, IV administration of VSV led to direct infection of ECs, triggering neutrophil infiltration and micro clot formation within tumor-associated vasculature, resulting in extensive bystander cell death [88]. Similarly, intravenous delivery of HSV in a murine ovarian carcinoma model showed selective infection and death of tumor-associated ECs without affecting normal ECs [89]. OVs can also serve as vectors for delivering angiogenesis inhibitors like bevacizumab, sorafenib, and sunitinib. Targeted expression of these inhibitors in the TME halts tumor growth and enables the viral progeny to spread through the tumor, infecting and lysing cancer cells to promote tumor clearance [87].

## Targeting tumor microenvironment

The TME is a multifaceted and ever-changing environment comprising different elements like immune cells, stromal cells, blood vessels, and the extracellular matrix (ECM). It plays a crucial role in cancer advancement by fostering tumor proliferation, infiltration, and spread. Rather than being inert, the TME actively promotes cancer progression through intricate interactions with cancer cells [90]. Immune cells present within the TME have the capacity to either hinder or facilitate tumor growth, with their impact varying depending on the individual health and other gene signatures that drive the disease complexity [91, 92]. Recent progress in computational analysis and modelling, utilizing data from single-cell transcriptomics, tumor expression profiles, has revealed variety of intercellular signalling networks within the TME [93]. One of the major hallmarks of cancer is the lack of oxygen, or hypoxia, and nutrients in certain regions of a growing solid tumor. The formation of new blood vessels via angiogenesis does not occur quickly enough to supply oxygen to the rapidly expanding tumor cells. As a result, some areas of the tumor become hypoxic, with oxygen levels dropping below 2% [94]. Tumor cells located within hypoxic regions exhibit heightened levels of hypoxia-inducible factor alpha (HIF-1α). HIFs are involved in tumorigenesis by regulating hypoxia-induced gene expression and metabolism [95]. HIF-1α boosts the activities of transcriptional factors like Twist and Snail, leading to increased endothelial-to-mesenchymal transition(EMT) [96]. Due to its role in collagen synthesis, collagen fibre alignment, and ECM interactions within the TME, HIF-1α facilitates tumor cell migration and metastasis [97]. Various molecules responding to hypoxia can initiate the angiogenic switch, with vascular endothelial growth factor (VEGF) and its downstream signaling pathway being the predominant drivers [98]. VEGF plays a central role in the TME by promoting the formation of new blood vessels from existing ones, which is crucial for tumor growth, invasion, and metastasis. VEGF is produced not only by tumor cells but also by other elements of the TME, such as tumor-associated macrophages and fibroblasts. Additionally, VEGF can modulate the TME by suppressing T cell activation [99, 100].

Beyond hypoxia and angiogenesis, the TME is a dynamic ecosystem where stromal cells and the ECM reciprocally influence tumor progression, metastasis, and therapy resistance. Stromal cells, including cancer-associated fibroblasts (CAFs), mesenchymal stem cells (MSCs), tumor-associated adipocytes (CAAs), tumor endothelial cells (TECs), and pericytes, originate from host tissues or via trans-differentiation and shape the TME. They secrete soluble factors such as cytokines (e.g., TGF-β, CXCL12), growth factors (e.g., VEGF, PDGF), and proteases that remodel the ECM, stimulate angiogenesis, EMT, and promote metastasis. CAFs and MSCs also release matrix metalloproteinases (MMPs) that degrade collagen and other ECM components, paving the way for tumor cell invasion and intravasation. These stromal cells can further reprogram tumor metabolism by altering nutrient availability, enabling cancer cells to thrive under hypoxic conditions. They also recruit immunosuppressive cells like myeloid-derived suppressor cells while inhibiting CTLs, thus aiding in immune escape [101, 102]. The ECM itself is not a passive scaffold but a complex network of proteins (like collagen and fibronectin), glycoproteins, and polysaccharides (such as hyaluronic acid) that regulate critical tumor behaviors. Increased ECM stiffness activates mechanosensitive pathways such as YAP/TAZ in tumor cells, driving EMT and chemoresistance. Additionally, interactions like hyaluronic acid binding to CD44 or HMMR receptors can promote EMT and metastasis. A dense ECM also impedes drug penetration and oxygen diffusion, which in turn leads to resistance against chemotherapy and radiotherapy. When combined, these complex interactions show how the TME produces an environment that is conducive to tumor growth and hinders effective treatment [91, 103]. This emphasizes how crucial it is to create therapeutic strategies that target or modify these intricate interactions.

Targeting the TME has become a critical focus in cancer therapy, as understanding how tumor cells interact with their surroundings is essential for developing effective treatments. Strategies to modulate the TME, such as immunotherapy and anti-angiogenic therapy, are being actively explored. However, despite its dynamic nature, our understanding of the specific variations the TME undergoes in human cancer remains limited. Cancer therapies, including radiotherapy, chemotherapy, targeted therapy, and immunotherapy, exert significant effects on the TME. For instance, chemotherapy can reduce neutrophil populations within the TME while influencing various aspects of tumor-associated macrophages, such as their recruitment, depletion, and phenotype regulation. Immunotherapy also impacts the TME, particularly affecting myeloid cells, though the extent of these effects on patient treatment responses is not yet fully understood. The complexity of the TME poses significant challenges to developing effective chemotherapeutic drugs. In this context, oncolytic virotherapy has emerged as a promising approach, offering the potential not only to directly eliminate tumor cells but also to modulate the immune response within the TME.

A crucial strategy in optimizing OVs for targeting the TME involves genetically engineering them to adapt to hypoxic conditions prevalent in tumors. One effective approach employs hypoxia-responsive promoters—genetic components that regulate the expression of viral genes under specific environmental conditions. These promoters are activated by HIFs, which are upregulated in oxygen-deprived regions of tumors. By linking critical viral gene expressions to HIFs, OVs can be engineered to preferentially replicate in hypoxic tumor areas. This strategy not only ensures precise targeting of tumor cells but also maximizes the virus’s therapeutic efficacy by enhancing its oncolytic activity in oxygen-deficient regions. Another genetic engineering technique involves the deletion of anti-apoptotic genes from the viral genome. By removing these genes, OVs exploit the dysregulated apoptotic pathways often present in cancer cells, increasing their vulnerability to viral lysis and replication. This selective replication ensures that OVs effectively kill cancer cells while sparing healthy tissues within the TME. In addition to genetic modifications, researchers are designing OVs to leverage hypoxia-induced pathways frequently activated in tumor cells under low-oxygen conditions.

For example, the HYPR-Ad series (Hypoxia-Promoted Replication Adenovirus) incorporates a bidirectional hypoxia/HIF-responsive promoter to simultaneously drive the expression of viral E1A genes essential for replication and therapeutic transgenes such as IL-4. This design enables the virus to replicate exclusively within hypoxic, HIF-active tumor cells, leading to immunogenic cell death and local IL-4 production that both stimulate anti-tumor immunity and exert anti-angiogenic effects. In preclinical models, HYPR-Ad demonstrated rapid tumor regression with efficacy comparable to the wild-type adenovirus dl309 [104]. Another approach, exemplified by AdLCY, employs a dual-regulated system combining six hypoxia response elements (HRE) responsive to HIF-2α and nine Oct4 response elements (ORE) activated by the stemness factor Oct4. This restricts viral replication to hypoxic bladder cancer cells co-expressing HIF-2α and Oct4, achieving superior tumor-selective oncolysis compared to viruses governed by a single promoter [105]. Meanwhile, Enadenotucirev (EnAd), a chimeric adenovirus, takes a different approach by downregulating HIF-1α during late-stage infection, thereby reducing VEGF and other pro-angiogenic factors, disrupting tumor vasculature, and enhancing T-cell infiltration into hypoxic regions. This systemic strategy has effectively reduced perfused vessels in xenograft models, amplifying the immunotherapeutic impact. Lastly, hypoxia-activated vaccinia viruses engineered with HRE-driven thymidine kinase (TK) or reporter genes like GFP demonstrate replication specifically within low-oxygen environments, targeting the hypoxic cores of solid tumors while sparing normal cells [106, 107].

By integrating these strategies, OVs are becoming increasingly effective tools for overcoming the challenges posed by the TME. Genetic engineering and pathway targeting allow OVs to selectively exploit the vulnerabilities of hypoxic tumors, offering a dual benefit of directly destroying cancer cells and modulating the immune system for long-term anti-cancer responses. As research progresses, these advancements hold the promise of transforming oncolytic virotherapy into a cornerstone of cancer treatment.

## Changes to look forward to—a translational approach

### Targeting cancer stem cells

A portion of cells residing within tumor structures exhibits resilience against traditional treatment methods and might be accountable for the recurrence of diseases. These cellular entities, termed as cancer stem cells (CSC), share characteristics with normal stem cells, including their capacity for self-renewal, pluripotency, resistance to drugs, and the ability to remain in a dormant state. Mainstream therapeutic approaches, primarily designed for rapidly dividing cells, are liable to overlook CSCs due to their slow division and prolonged quiescence [108]. The surviving CSCs, with their capability for self-renewal and differentiation, can instigate a relapse of the disease following an initial remission. In contrast to conventional treatments, OVs possess the potential to eliminate both differentiated cells and CSCs, offering the prospect of disease eradication. The resistance of CSCs to conventional therapies is attributed to their elevated expression of multi-drug resistance, heightened DNA repair proficiency, and their capacity to endure extended quiescence. However, OVs remain unaffected by these characteristics, enabling them to replicate within CSCs and induce the lysis of these cells. In a study conducted in 2007, Eriksson et al. identified CSCs (CD44 + CD24-/low) from breast cancer patients’ pleural effusions and assessed two oncolytic adenoviruses, namely Ad5/3-Δ24 and Ad5.pk7-Δ24. Both OVs demonstrated potent efficacy in vitro, causing the death of CSCs. Furthermore, the infection of CSCs with either of these viruses negated the tumor-forming capacity of those CSCs in SCID mice [109]. Another investigation by Yoo et al. revealed the susceptibility of drug-resistant CSCs (CD133 + CD44+) from human colon cancer cell lines to an oncolytic vaccinia virus, contrasting their resistance to the drug CPT11 [110]. Oncolytic herpes simplex virus type 1 (oHSV-1) has demonstrated significant anticancer effects against glioblastoma stem cells (GBM-SCs), reducing secondary tumorsphere formation and extending survival in mouse models of aggressive GBM [111]. Engineered strains of measles virus, such as MV-141.7 and MV-AC133, specifically target CD133-positive CSCs, leading to precise eradication of these tumor-initiating cells [112]. Similarly, oncolytic vaccinia virus (VACV) strains like GLV-1h68 preferentially replicate in tumor cells with high Aldehyde dehydrogenase 1(ALDH1) activity, a marker of stem-like properties, allowing selective destruction of CSCs while sparing normal stem cells [113]. Modified oncolytic adenoviruses (ADVs), such as Ad-VT, have been engineered to induce apoptosis in breast CSCs by targeting key survival pathways [114]. Furthermore, clinical trials with DNX-2401, a genetically engineered ADV, in combination with immune checkpoint inhibitors like pembrolizumab, have been explored to enhance therapeutic efficacy in gliomas, which are enriched in CSC populations [115]. Collectively, these strategies highlight the versatility of oncolytic virotherapy in overcoming CSC-mediated tumor progression.

### Zika virus targets glioblastoma stem cells

**Fig. 5:**
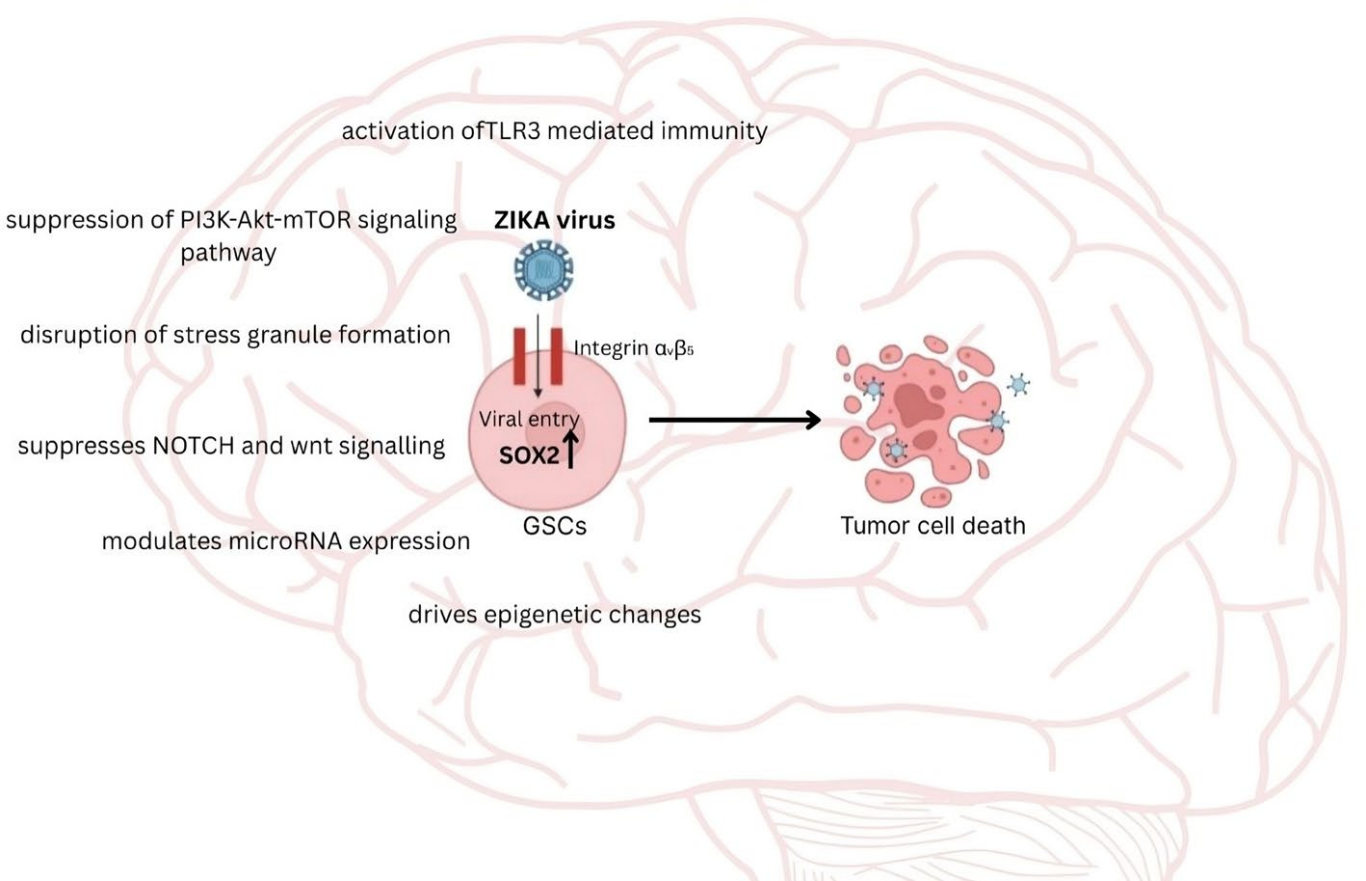
ZIKV selectively targets GSCs primarily through the SOX2–Integrin αvβ5 axis, facilitating efficient viral entry and replication. In addition to this targeted entry mechanism, ZIKV activates TLR3-mediated innate immune responses, suppresses the PI3K-Akt-mTOR signaling pathway, disrupts stress granule assembly, inhibits Notch and Wnt signaling pathways, modulates microRNA expression, and induces epigenetic reprogramming. Collectively, these alterations compromise the survival and stemness of GSCs, leading to their apoptosis and ultimately contributing to the suppression of GBM progression

GBM is one of the deadliest human cancers, with existing treatments providing only limited relief rather than a cure. OVs, including Zika Virus (ZIKV), offer a promising novel approach for treating GBM. ZIKV stands out as a compelling candidate due to its unique ability to selectively infect and eliminate Glioblastoma stem cells (GSCs) (Fig. 5).

ZIKV selectively targets GSCs through multiple molecular mechanisms, most notably the SOX2–Integrin αvβ5 axis. GSCs are characterized by high expression of SOX2, a transcription factor critical for maintaining their stemness and self-renewal capacity. SOX2 upregulates the expression of Integrin αvβ5, a cell surface receptor involved in adhesion and signalling. This integrin also serves as a co-receptor for ZIKV entry, allowing the virus to bind and enter GSCs more efficiently than differentiated tumor cells or normal brain cells, which have lower levels of both SOX2 and integrin αvβ5. Once inside, ZIKV replicates robustly within GSCs, leading to their selective death [116]. Beyond this axis, ZIKV also activates TLR3-mediated innate immunity, inducing apoptosis; disrupts cell cycle progression [117], causing mitotic catastrophe; and suppresses the PI3K-Akt-mTOR signalling pathway, which is essential for cell growth, survival, and maintenance of stemness. Notably, ZIKV’s NS4A and NS4B proteins have been shown to inhibit this pathway, resulting in aberrant activation of autophagy and disruption of neurogenesis [118], further contributing to cytotoxicity in GSCs. The virus also disrupts stress granule formation, which normally acts as a protective mechanism by halting mRNA translation and sequestering pro-apoptotic signals during cellular stress. ZIKV achieves this through the actions of its NS3 and NS5 proteins, which interfere with key granule-nucleating proteins such as G3BP1 and TIA-1, thereby preventing the assembly of stress granules. This renders GSCs more vulnerable to viral replication-induced stress and apoptosis [119]. Additionally, suppresses Notch and Wnt signalling [120], and modulates microRNA expression to promote cell death. Additionally, ZIKV’s NS5 protein blocks interferon responses and alters host gene transcription, while infection-driven epigenetic changes weaken the tumor’s regenerative capacity. Together, these pathways enable ZIKV to act as a potent and selective oncolytic agent that targets therapy-resistant GSCs while sparing normal brain tissue. ZIKV has demonstrated significant antitumor effects across various glioblastoma and central nervous system (CNS) tumor models. In a human case report, a glioblastoma patient who developed a post-surgical arbovirus-like infection during the ZIKV outbreak showed complete tumor regression and remained recurrence-free for six years, suggesting a potential natural oncolytic effect of ZIKV or a related flavivirus [121]. Similarly, in orthotopic mouse models of aggressive embryonal CNS tumors (e.g., medulloblastoma, ATRT), both intracranial and systemic ZIKV administration resulted in significant tumor regression and prolonged survival, with histopathological evidence of tumor clearance and no severe side effects. A companion human organoid co-culture model confirmed reduced tumor progression and elevated inflammatory cytokine levels (MIF, Tnfsf13b) following ZIKV exposure [122]. In a canine study involving three dogs with spontaneous CNS tumors, intrathecal injection of ZIKV led to MRI-confirmed tumor shrinkage, dramatic clinical improvement, and clearance of viral RNA within 14 days, without adverse effects [123]. Additionally, in a GSC model, ZIKV infection induced miR-34c expression, triggering apoptosis and growth arrest of GSCs in vitro and replicating these therapeutic effects in vivo [124]. Notably, a Phase I clinical trial (NCT05123222) evaluating the safety and immunogenicity of two ZIKV strains in healthy adults was completed on September 3, 2024, marking a key step toward clinical translation of ZIKV-based interventions [125]. These findings collectively underscore ZIKV’s potent and selective oncolytic activity against therapy-resistant CNS tumors, supporting its continued development as an innovative virotherapeutic agent.

### Poxvirus mediated oncolytic therapy

The prototypical member of the Poxviridae family’s Leporipoxvirus genus is Myxoma virus (MYXV), a DNA virus. In its natural state, MYXV exhibits an exceptionally limited host range, predominantly affecting European rabbits and causing the rabbit-specific ailment myxomatosis. Other tested host species, including humans, do not manifest any discernible disease upon MYXV infection. This suggests its viability as an exceptionally secure OV for cancer patients [126]. An investigation revealed that pre-treatment with replication competent MYXV before cisplatin administration significantly amplifies the therapeutic advantages of chemotherapy [127]. MYXV boasts notable immunomodulatory effects and a predilection for malignant cells, presenting considerable potential for evolving into a cancer-fighting therapeutic approach. Cancer cells with defective interferon pathways are highly permissive to myxoma virus, which is otherwise attenuated in normal cells. This potential can be further realized through diverse genetic alterations aimed at augmenting its oncolytic efficacy while mitigating its pathogenicity.

### Non-infectious PVNPs enhance immune response

Immunotherapy using nanoparticles is an emerging area in oncology with considerable therapeutic potential. While much of the existing research has centered on nanoparticles (NPs) as delivery vehicles for chemotherapeutics, their inherent tendency to be taken up by innate immune cells positions them as promising immunomodulatory agents. Among biologically derived NPs, plant viruses (PVs) are especially attractive due to their biosafety profile—being non-infectious to humans and unable to replicate in mammalian systems. Their proteinaceous capsids display PAMPs that are recognized by pattern recognition receptors (PRRs), leading to activation of innate immunity and reprogramming of the TME [128]. A specialized class of PVs, plant-virus-based nanoparticles (PVNPs), has gained attention for their dual role in drug delivery and immune activation. These are broadly categorized into virus-like particles (VLPs), which are genome-free and non-infectious, and virion nanoparticles (VNPs), which contain native viral RNA but remain non-replicative in mammals. VLPs offer enhanced safety, whereas VNPs may exhibit additional immunogenic effects due to their nucleic acid content. Despite advances in nanocarrier technology, systemic delivery of NPs results in minimal accumulation at tumor sites (~ 1%), even under enhanced permeability and retention (EPR) conditions, necessitating more efficient delivery strategies. PVNPs meet this need by serving as both cargo carriers and immune activators, capable of inducing immunogenic cell death, enhancing immune cell infiltration, and reshaping the TME [129, 130]. Table 4 summarizes representative examples of PVNPs, highlighting their structure, anticancer mechanisms, tumor models used, and key therapeutic outcomes.

**Table 4:** Comparison of plant viral oncotherapy under study

Plant Virus	Structure Type	Mode of Anticancer Action	Cancer Model	Key Findings	References
Cowpea Mosaic Virus (CPMV)	VLP (Empty Virion)	Acts as an in-situ vaccine by activating neutrophils and adaptive immunity; disrupts tumor immunosuppression and induces systemic anti-tumor response	B16F10 melanoma, 4T1 breast, CT26 colon, ID8 ovarian carcinoma	Inhaled eCPMV suppresses lung melanoma and induces systemic immunity against metastases; effective across multiple tumor types.	[128]
Tobacco Mosaic Virus (TMV)	Rod-shaped virion (300 × 18 nm nanotube)	Delivers pre-activated cisplatin (cisPt²⁺) directly to tumor via EPR; mild innate immune stimulation in immunocompetent hosts	Ovarian cancer (ID8-Defb29/Vegf-a-Luc; A2780 xenograft)	TMV-cisPt accumulates in tumors, enhances efficacy over free cisplatin, reduces tumor burden, and extends survival; safe and well tolerated. Empty TMV shows mild antitumor effects in immunocompetent mice.	[131]
Potato Virus X (PVX)	Filamentous virion (515 × 13 nm)	Passive targeting via EPR; carries doxorubicin through hydrophobic interactions; enhances tumor penetration	MDA-MB-231 (breast), A2780 (ovarian), HeLa (cervical)	PVX-DOX retains drug potency, improves tumor accumulation, reduces tumor burden in MDA-MB-231 xenografts; PEGylation enhances circulation stability.	[132]
Papaya Mosaic Virus (PapMV)	VLP (rod-shaped, ~ 80 nm)	Activates innate immunity via TLR7; induces IFN-α; reduces MDSCs; boosts CD8⁺ T cell responses; acts as in situ adjuvant	B16F10 and B16-OVA melanoma (s.c. and metastatic models)	PapMV delays tumor growth, reduces metastasis, increases tumor-specific CD8⁺ T cells, and synergizes with PD-1 blockade and DC-based vaccines; promotes immunological memory.	[133]
Cowpea Chlorotic Mottle Virus (CCMV)	VLP (poly(I: C)-loaded capsid)	Delivers TLR3 agonist (poly(I: C)) to activate innate/adaptive immunity; enhanced efficacy in combination with oxaliplatin	CT26 colon carcinoma (i.p. and s.c. murine models)	CCMV-poly(I: C) activates macrophages, boosts cytokine production (IFN-γ, IL-4), increases CD4⁺/CD8⁺ infiltration; combined with oxaliplatin enhances apoptosis, ICD, and survival; shows synergistic immune–chemo response.	[134]

## Synergistic allies of tumor-targeting virotherapeutics—combinatorial therapies

### Radiotherapy and tumoricidal viruses

Radiation therapy contributes to approximately 40% of all cancer cures globally [135]. Advances in radiotherapy techniques have led to reduced toxicity due to increased precision and the ability to modulate radiation delivery. OVs and radiotherapy are two distinct cancer treatment modalities with different mechanisms and non-overlapping cytotoxicity profiles. Therefore, the potential for OVs to function as cancer-selective radiosensitizers is an intriguing possibility. This approach could enhance the effects of radiation treatment on tumors while having minimal impact on normal tissue. Adenoviruses have evolved various interactions with cellular DNA damage repair proteins to facilitate viral replication. This interaction influences the initiation of several DNA repair pathways activated in response to radiation-induced damage. Notably, all adenoviral serotypes tested appear to target the non-homologous end joining (NHEJ) repair pathway [136]. The hypothesis that oncolytic adenovirus infection could work synergistically with radiotherapy has been explored by multiple research groups. CG7870 is a replication-selective oncolytic adenovirus genetically engineered to replicate preferentially in prostate tissue. Combining CG7870 with radiation resulted in a synergistic increase in cell death, both in vitro and in vivo in xenograft model, compared to either treatment alone [137]. Oncolytic vaccinia virus with F4L and J2R gene deletions (ΔF4LΔJ2R VACV) demonstrated effective replication and cytotoxicity in radiation-treated brain tumor initiating cells under laboratory conditions. Notably, when comparing therapeutic approaches in immune-competent orthotopic CT2A-luc mouse models, the combination of a single 10 Gy radiation dose with ΔF4LΔJ2R VACV treatment yielded remarkably superior anti-tumor outcomes compared to either therapy alone. This combinatorial approach significantly prolonged survival and achieved complete remission in most experimental animals. Furthermore, mice that recovered following this combined treatment exhibited substantially enhanced survival rates compared to untreated age-matched control animals upon subsequent intracranial tumor challenge, with several animals demonstrating complete tumor rejection. These findings suggest that the radiation-virus combination therapy not only effectively eliminates existing tumors but also potentially establishes durable anti-tumor immunity that protects against future recurrence [138].

### Chemotherapy and virotherapy

Chemotherapy drugs primarily target actively dividing cells by disrupting DNA replication and the cell cycle. While this approach is highly effective against cancer cells, it can also negatively impact rapidly proliferating healthy cells. In contrast, OVs selectively infect and destroy tumor cells, minimizing damage to normal tissues. The combination of chemotherapy’s cytotoxic effects with the tumor specificity of OVs may result in a synergistic therapeutic outcome. One notable example is H101, which was approved in China in 2005 for the treatment of nasopharyngeal carcinoma in combination with chemotherapy [47]. Additionally, a Phase 2 clinical trial evaluated the combination of T-VEC with neoadjuvant chemotherapy (NAC) for the treatment of triple-negative breast cancer. The trial successfully met its predefined primary efficacy endpoint, demonstrating that T-VEC in combination with NAC may significantly enhance pathologic complete response (pCR) rates compared to chemotherapy alone. These findings underscore the potential of combining chemotherapy and oncolytic virotherapy to improve treatment efficacy, offering a promising approach for enhanced anti-cancer outcomes [139].

### Oncolytic viruses and checkpoint inhibitors

Immune checkpoints (ICIs) are key regulators of the immune system, playing a crucial role in maintaining self-tolerance and preventing indiscriminate attacks on cells by the immune system [140]. Many tumors manage to evade the host immune response by upregulating these checkpoints to create an immunosuppressive TME [141]. ICIs function by disrupting the tumor’s immunosuppressive signalling pathways, thereby exposing cancer cells to the host immune system. DNX2401 is an oncolytic adenovirus that exhibits enhanced infectivity and tumor specificity [142]. This improvement is due to a 24-base pair deletion in the Rb binding site of the E1A region, allowing selective replication in cancer cells lacking a functional Rb pathway [143]. DNX2401 has shown promising results in preclinical studies and is currently being evaluated in three clinical trials for treating malignant gliomas. Following intra-tumoral injection, the vector led to reduction of tumor size in 72% of patients, with a median overall survival of 9.5 months. Of particular interest is Lang et al.‘s finding of decreased TIM-3 expression through immunohistochemical analysis of resected tumor specimens [144]. TIM-3 is an immune inhibitory receptor associated with T cell exhaustion [145]. DNX2401 infection may mitigate some aspects of T cell exhaustion in glioma patients, providing a basis for investigating the vector in combination with anti-PD-1 antibodies [144]. A phase II trial is underway to assess DNX2401 administered intratumorally, followed by the PD-1 inhibitor pembrolizumab, in patients with recurrent glioblastoma. Interim results for 48 patients who received the combination showed a median overall survival of 12 months, with 47% experiencing clinical benefits (stable disease or better). Notably, four patients had partial responses, two of whom showed a 94% reduction in tumor volume, and three survived for more than 20 months [146]. ONCOS-102 is a highly modified oncolytic adenovirus vector featuring a a 24-base pair deletion in the E1A region for selective replication in cells with a dysfunctional Rb pathway, and a GM-CSF transgene to enhance immune cell infiltration at the tumor site [147, 148]. ONCOS-102 has been extensively evaluated in preclinical studies and has advanced to a phase I clinical trial in combination with the PD-1 ICI pembrolizumab. In this trial, 12 patients with advanced or unresectable solid tumors were treated, demonstrating that ONCOS-102 is safe and well-tolerated at tested doses, with the combination therapy inducing significant immune cell infiltration at tumor sites. The study found that combination therapy led to 5.9- and 4.0-fold increases in CD3 + immune cells and CD8 + T cells infiltration, respectively, in post-treatment tumor biopsies compared to pre-treatment samples [148]. Notably, two of the 12 patients exhibited increased PD-L1 expression following ONCOS-102 administration and developed systemic anti-tumor immunity, as evidenced by melanoma-associated antigen 3 (MAGE-A3)-specific CD8 + T cells and New York oesophageal squamous cell carcinoma 1 (NY-ESO-1)-specific CD8 + T cells [149]. MAGE-A3 and NY-ESO-1 are both antigens that are associated with cancer, and both can be targeted by CD8 + T cells and NY-ESO-1 expression is associated with a worse prognosis. Based on these observations, another phase I clinical trial has been initiated to test ONCOS-102 with pembrolizumab in recurrent patients after PD-1 blockade [150], thus demonstrating the combined treatment potential of ICIs and OVs.

### Overcoming challenges in combinatorial therapies

Combining oncolytic virotherapy with standard cancer treatments such as radiotherapy, chemotherapy, and ICIs presents several therapeutic challenges. Radiotherapy and OVs require precise scheduling and dose calibration to avoid compromising viral replication or tumor specificity, and translation from preclinical to clinical settings remains limited. Patient immune status and the narrow therapeutic window also impact efficacy. These issues may be addressed through dose-optimization studies, the use of radiation-inducible viral promoters, and adaptive clinical trial designs [137]. When combined with chemotherapy, OVs face challenges such as overlapping toxicities, reduced viral replication due to immunosuppression, and poor tumor penetration. Solutions include metronomic dosing to preserve immune function, timing-based regimens, and protective delivery systems like capsid shielding or cellular carriers, with biomarker-guided approaches aiding schedule refinement [139]. Similarly, integrating ICIs with OVs is hindered by the immunosuppressive TME, premature antiviral immune clearance, and increased risk of immune-related toxicity. These can be mitigated by arming OVs with immunostimulatory cytokines (e.g., GM-CSF, IL-12), sequencing therapy to support viral replication and T-cell priming, and employing intratumoral delivery or carrier cells to enhance tumor localization [146]. Collectively, these integrated strategies aim to overcome mechanistic barriers and harness the synergistic potential of combination regimens to enhance tumor control and patient outcomes.

## Directed evolution: revolutionizing oncolytic virus design for precision cancer therapy

Directed evolution synthesis is an innovative strategy for optimizing OVs. Inspired by natural evolutionary processes, this technique accelerates the development of OVs by mimicking adaptive changes that enhance their therapeutic performance. In traditional approaches, OVs are often genetically engineered based on existing knowledge of viral genetics and cancer biology. In contrast, directed evolution circumvents the constraints of rational design by applying selective pressure in controlled environments, facilitating the emergence of viral variants with improved tumor specificity and immune activation. The directed evolution process typically begins with a diverse viral library, which can include naturally occurring viruses or genetically modified variants. This library is exposed to specific conditions that mimic the TME, such as hypoxia, acidic pH, and immune components, allowing the viruses to adapt and evolve traits that enhance their ability to thrive in these conditions. Over multiple rounds of replication, selection, and amplification, viral populations that demonstrate improved characteristics, such as higher replication rates, enhanced oncolytic activity, or better immune evasion, are preferentially enriched. These optimized variants are then isolated and characterized to identify the genetic and phenotypic changes responsible for their improved performance. One of the key advantages of directed evolution is its ability to uncover novel mutations or genetic combinations that might not have been predicted through rational design. This unbiased approach enables researchers to discover new pathways or mechanisms by which OVs can selectively target cancer cells. For example, viruses evolved under hypoxic conditions may develop mutations that allow them to exploit HIFs, enhancing their replication in oxygen-deprived tumor regions. Similarly, directed evolution can lead to the selection of viral variants with enhanced tropism, enabling them to bind more effectively to receptors overexpressed on cancer cells. The iterative nature of directed evolution also makes it particularly suitable for optimizing OVs for use in diverse and complex TMEs [151, 152].

Examples include oloAd1, a next-generation oncolytic adenovirus created by pooling multiple adenovirus serotypes followed by extensive in vitro selection. This virus demonstrated 100–1,000 times greater potency than ONYX-015, with selective replication in colorectal cancer cells. Similarly, NGOVM, evolved through serial passaging in HCT-116 colon cancer cells, acquired mutations (notably in the E2 and nsP3 genes) that conferred a 9,690-fold increase in oncolytic activity and expanded tropism across various solid tumors. These adaptations arose from the viruses’ ability to overcome intracellular antiviral defenses and exploit the unique metabolic environment of cancer cells [152, 153].

Recently, researchers utilized this approach to engineer OVs with improved tumor-targeting abilities and immune-stimulatory effects [152]. The directed evolution synthesis of OVs represents a paradigm shift in oncolytic virotherapy, combining the power of natural selection with modern molecular biology techniques. This method not only accelerates the development of highly effective OVs but also broadens the scope of their application across various cancer types. As the field advances, integrating directed evolution with other cutting-edge technologies, such as high-throughput screening and computational modelling, will likely yield even more sophisticated and potent OVs for clinical use, paving the way for transformative breakthroughs in cancer treatment.

## Safety profile and bioethical considerations

Since OVs are self-replicating in nature, careful consideration is needed to address the risk of unintentional transmission from treated patients to close contacts and the surrounding environment. OVs are typically engineered or selected to ensure they do not infect or replicate in normal cells, minimizing the risk of transmission to unintended individuals. Ensuring proper handling of infectious materials and maintaining biological safety is crucial when administering an OV. It is essential to follow established biosafety guidelines or their equivalent while strictly adhering to all relevant institutional, national, state, and local regulations.

As of today, there have been no documented cases of transmission to healthcare professionals or caregivers. In most clinical trials that assessed viral shedding using infectivity tests—either alone or combined with PCR—a few studies did report the presence of infectious viral particles in some samples. For instance, in trials involving the oncolytic adenovirus CG7870, saliva samples from metastatic prostate cancer patients treated with high doses revealed infectious particles. Similarly, in trials with the oncolytic adenovirus ONCOS-102, three patients with solid tumors who received repeated intratumoral injections had detectable infectious viral particles in buccal samples and in urine [148]. In patients treated with the Herpesvirus Imlygic^®^ for recurrent melanoma, swabs from the surface of injected lesions tested positive for viral infectivity [154]. Although shedding of infectious viral particles during oncolytic virotherapy appears to be a relatively rare event, these observations highlight the importance of thorough monitoring. Despite the low risk, concerns about viral shedding still exist, particularly for immunosuppressed patients, as well as the possibility of OVs with recombinant DNA recombining with wild-type viruses.

Adverse events (AEs) associated with oncolytic virotherapy are generally mild to moderate in severity, with patients experiencing manageable side effects. The frequently reported AEs resemble flu-like symptoms, including fever, chills, fatigue, muscle aches, headache, and malaise, which usually arise within the first few days of treatment and subsides within a few days [155]. Local reactions at the injection site are also common. Gastrointestinal disturbances like nausea, vomiting, and diarrhea may occur but are usually transient. Fatigue is particularly notable, affecting more than half of treated patients. Less common or more severe AEs include mild, transient elevations in liver enzymes, especially with adenovirus-based OVs, and sporadic occurrences of hypotension, mild hypoxemia, arthralgia, and dizziness. Although rare, serious grade 3 to 4 AEs have been documented, such as hyponatremia, dyspnea, pleural effusion, hypoalbuminemia, anemia, dehydration, and cerebral edema. Immune-related events like cytokine release syndrome and transient mild encephalopathy have also been reported, but significant neurotoxicity is uncommon [156]. Fortunately, most AEs associated with OV therapy are self-limiting and can be managed with supportive measures such as antipyretics and analgesics. However, persistent or serious AEs warrant prompt medical evaluation and may require discontinuation of therapy or additional interventions.

A recent example highlights the intense ethical nuances involved with oncolytic virotherapy. In this case, a breast cancer patient who had recurrent disease chose to administer laboratory-grown OVs herself intratumorally, choosing to avoid another round of chemotherapy. She used two viruses in succession, measles virus followed by VSV, both of which have shown specificity for her tumor type and have previously been tested clinically. Four years after this unusual treatment she is still cancer-free. Although this result is certainly intriguing, it raises significant ethical and safety implications regarding the unsupervised application of experimental treatments. These self-initiated interventions bypass clinical regulation, strict safety monitoring, and ethical review procedures intended to safeguard patients and the health of the general public. This case is a stark reminder of the need for robust biosafety protocols, rigorous ethical guidelines, and professional oversight to inform the safe development and use of oncolytic virotherapy [157].

## Addressing hurdles and strategies for success in oncolytic virotherapy

Several trials investigating diverse OVs, including HSV, adenovirus, and others, have demonstrated encouraging outcomes (Table 5). These trials have highlighted the relative safety profile of OVs and their potential to induce tumor regression. Notably, some studies have reported durable responses and even complete remissions.

**Table 5:** Promising oncolytic viruses from completed clinical trials in cancer therapy

Virus	Clinical Trial Number	Phase/Status	Cancer	Reference
DNX-2401	NCT00805376	I /Completed	Recurrent malignant glioma	[158]
PVSRIPO	NCT01491893	I Completed	Recurrent malignant glioma	[159]
Ad-RTS-hIL-12 + VDX	NCT02026271	I Completed	Recurrent/progressive GBM or grade III malignant glioma	[160]
Ad-RTS-hIL12 + VDX + Nivolumab	NCT03636477	I Completed	Recurrent or progressive GBM	[161]
JX-594	NCT026330368	I/II Completed	Advanced BC and sarcoma	[162]

The problem of non-specific targeting, in turn adversely impacting normal cells, is the main challenge in the clinical use of OVs. The adenovirus Ad5/3-Δ24 is genetically modified to interact with integrins exhibiting on ovarian cancer cells at significantly high concentrations and are now under clinical trials [163]. Similarly, the measles virus has been modified to express a single-chain antibody that specifically targets carcinoembryonic antigen (CEA), a tumor-associated marker found in certain human adenocarcinomas [6].

Another drawback with OVs is, that the patients may be immune to the virus itself due to past natural exposures or through vaccinations. A common strategy to reduce the neutralization of the virus involves employing different virus serotypes. Both adenoviruses and VSV have multiple serotypes, enabling the switching of serotypes between injections to thwart antibody neutralization. For example, in adenoviruses, high seroprevalence of neutralizing antibodies against common serotypes like Ad5 is a significant challenge, as these antibodies can bind to viral capsid proteins and block infection of target tumor cells. To address this, researchers employ pseudo-typing approaches—replacing the Ad5 fiber protein with those from less common serotypes such as Ad45 or Ad35. For instance, an Ad5 vector pseudo-typed with Ad45 fiber (Ad5/f45) demonstrated remarkable resistance to neutralization by sera rich in anti-Ad5 antibodies, thereby maintaining its capacity to transduce target cells even in the presence of pre-existing immunity. This approach takes advantage of the natural serotype diversity within adenoviruses; less prevalent serotypes like Ad35 encounter fewer neutralizing antibodies in the general population, making them valuable for designing vectors with improved systemic delivery profiles [164, 165]. A similar strategy applies to VSV, which exists in multiple serotypes, notably the Indiana (VSV-Ind) and New Jersey (VSV-NJ) serotypes. Antibody responses generated against one serotype often do not fully cross-neutralize another, due to differences in glycoprotein structure. This adaptability is enhanced by the ease with which VSV vectors can be engineered to express glycoproteins from alternative serotypes, providing a flexible system for sequential treatments [166, 167]. One of the major hurdles in oncolytic virotherapy is therapy resistance, where tumors either fail to respond or develop mechanisms to evade viral infection and lysis. Resistance can arise at multiple levels: intrinsic cellular resistance due to defective viral entry receptors or antiviral signalling (e.g., intact interferon pathways); TME-mediated resistance, such as immunosuppressive cytokines, dense extracellular matrix, or limited viral spread; and adaptive resistance resulting from immune clearance of the virus before it can exert therapeutic effects. To overcome these barriers, several strategies are being developed. Arming OVs with immunomodulatory transgenes (e.g., IL-12, GM-CSF, anti-PD-L1 antibodies) can reprogram the TME and sustain immune pressure on tumors. Retargeting viral tropism through capsid engineering or synthetic ligands allows OVs to infect resistant tumor cells more efficiently. Combination therapies with ICIs, radiation, or epigenetic drugs can sensitize tumors to viral infection and boost antitumor immunity. Sequential or priming strategies, where one OV is used to condition the TME before delivering a second therapeutic virus, are also being explored. Finally, patient stratification using predictive biomarkers (e.g., IFN pathway mutations, integrin expression) can help identify tumors likely to respond, enabling a more personalized and effective application of virotherapy.

Furthermore, to mitigate the risk of recombination with wild-type viruses, which could potentially generate more pathogenic or less controllable strains, multiple safeguards are employed. Genetic engineering strategies such as deleting essential genes for replication in normal cells (e.g., γ34.5 in HSV-1) restrict replication to tumor tissues and reduce opportunities for recombination. Incorporating safety switches, like retaining thymidine kinase genes that confer sensitivity to antivirals (e.g., acyclovir for HSV-based OVs), allows rapid intervention if unintended replication occurs. Using viral strains with low human pathogenicity and limited environmental reservoirs (e.g., NDV, VSV, SVV) further minimizes risks. Rigorous containment and biosafety protocols, including handling under BSL-2 or ABSL-2 conditions and monitoring for viral shedding, limit environmental release and exposure to vulnerable individuals [168]. Additionally, engineering viruses with unique sequences that have low homology with circulating wild-type viruses reduces recombination chances. Routine screening for replication-competent viruses and advising patients to avoid exposure to wild-type viral environments during therapy add further layers of safety. Together, these strategies form a framework to mitigate recombination risks while maintaining the therapeutic benefits of oncolytic virotherapy.

## Conclusion

Oncolytic virotherapy is a promising new approach to cancer treatment. This review has shown that OVs can selectively target and destroy cancer cells while also triggering an immune response against the tumor. OVs work in multiple ways, and scientists are developing methods to engineer viruses for enhanced effectiveness against cancer. Oncolytic virotherapy can also be combined with other cancer treatments, such as immunotherapy, to improve outcomes. The capacity of OVs to induce localized inflammation, serve as gene delivery vectors, and directly destroy tumors makes them suitable candidates for strategic combinations in therapy. The interactions between viruses and our immune systems offer numerous prospects for combining OV therapy with ICIs and/or adoptive cell therapy (ACT). However, there are still challenges to overcome, such as developing strategies to prevent treatment resistance and determine the optimal dosing schedule. As researchers learn more about oncolytic virotherapy, it is becoming clear that this approach has the potential to revolutionize cancer treatment and offer new hope to patients.

## Future perspectives

The field of oncolytic virotherapy has made remarkable strides, becoming a promising approach for advanced cancers, especially those unresponsive to standard treatments. Since the FDA’s approval of T-VEC in 2015, there has been a surge in clinical research involving various viruses such as vaccinia, reovirus, parvovirus, and picornavirus. As of July 1, 2025, a total of 220 clinical trials investigating OVs for cancer treatment are registered on ClinicalTrials.gov (https://clinicaltrials.gov/), reflecting the growing clinical interest in this therapeutic approach. These include seventeen early Phase I trials, one hundred fifty-four Phase I trials, and seventy-nine Phase II trials, indicating that the majority of studies are focused on establishing safety, dosing, and preliminary efficacy. Notably, there are five Phase III trials and two Phase IV trials, signaling that a select number of OV-based therapies are progressing toward late-stage validation and post-marketing evaluation. This distribution underscores the advancing translational potential of OVs from experimental platforms to viable clinical cancer therapies [169]. These viruses are particularly effective in combination with conventional therapies like chemoradiotherapy, demonstrating enhanced tumor reduction and improved patient outcomes. Oncolytic virotherapy holds great promise, but its clinical translation is hindered by challenges such as delivery barriers, immune clearance, and manufacturing limitations. Systemic administration often leads to rapid viral neutralization and poor tumor penetration, while pre-existing immunity can reduce viral replication before therapeutic effects are achieved. Additionally, large-scale manufacturing remains complex due to the need for live-cell production systems, biosafety containment, and batch variability. Addressing these issues will require integrated solutions: improved delivery strategies (e.g., cell-based carriers, nanoparticle encapsulation), capsid modifications to evade immunity, and optimized treatment scheduling. To overcome manufacturing and scalability barriers, efforts are focusing on using standardized producer cell lines, suspension bioreactors, serum-free media, and continuous downstream purification systems. Advances in synthetic biology are enabling the design of modular, high-yield viral genomes tailored for both efficacy and production. Looking ahead, the development of personalized virotherapy platforms, guided by tumor and immune profiling, and AI-driven treatment optimization will pave the way for safer, scalable, and more effective oncolytic virotherapy.

## Data Availability

No datasets were generated or analysed during the current study.

## Abbreviations

OVs: Oncolytic Viruses
PVNPs: Plant Virus-Based Nanoparticles
VNPs: Virion Nanoparticles
VLPs: Virus-Like Particles
GSCs: Glioblastoma Stem Cells
ZIKV: Zika Virus
GBS: Guillain-Barré Syndrome
ISGs: Interferon-Stimulated Genes
TNFα: Tumor Necrosis Factor Alpha
TRAIL: Tumor Necrosis Factor-Related Apoptosis-Inducing Ligand
GBM: Glioblastoma
CSCs: Cancer Stem Cells
NDV: Newcastle Disease Virus
IFN: Interferon
DCs: Dendritic Cells
IFN-α: Interferon-Alpha
IFN-β: Interferon-Beta
JAK: Janus Kinase
STAT: Signal Transducer and Activator of Transcription
PKR: Protein Kinase R
IT: Intra-Tumor
IV: Intravenous
IM: Intramuscular
NPs: Nanoparticles
TME: Tumor Microenvironment
EPR: Enhanced Permeability and Retention
MYXV: Myxoma Virus
APMV-1: Avian Paramyxovirus Type 1 Virus
NO: Nitric Oxide
ACT: Adoptive Cell Therapy
CEA: Carcinoembryonic Antigen
